# Editorial: Understanding Protein Dynamics, Binding and Allostery for Drug Design

**DOI:** 10.3389/fmolb.2021.681364

**Published:** 2021-04-21

**Authors:** Guang Hu, Pemra Doruker, Hongchun Li, Ebru Demet Akten

**Affiliations:** ^1^Center for Systems Biology, Department of Bioinformatics, School of Biology and Basic Medical Sciences, Soochow University, Suzhou, China; ^2^Department of Computational and Systems Biology, School of Medicine, University of Pittsburgh, Pittsburgh, PA, United States; ^3^Research Center for Computer-Aided Drug Discovery, Shenzhen Institutes of Advanced Technology, Chinese Academy of Sciences, Shenzhen, China; ^4^Department of Bioinformatics and Genetics, Faculty of Engineering and Natural Sciences, Kadir Has University, Istanbul, Turkey

**Keywords:** allostery, drug discovery, molecular dynamics simulation, elastic network model, protein structure network

Proteins as molecular machines have dynamic structures sampling various conformational states, which determine their functionality, ligand binding, and allosteric properties. Allosteric communication as an intrinsic property of proteins (Gunesakaran et al., 2004) can be triggered by physical (protein-protein interaction, ligand-binding) and chemical (mutations, post-translational modification) events happening distant from the orthosteric site (Zhang et al., 2020). Protein dynamics and conformational transitions govern allosteric communication between distinct sites, which is central for the regulation of protein function, signal transduction, and approaches in drug discovery.

Computational modeling and simulations at hierarchical levels of complexity have become requisite for unraveling the link between protein structure, dynamics and function, as well as toward designing agents that regulate protein function. Being an active research field for over a century, allostery has become pivotal today in the pursuit of designing allosteric modulators with specificity. Simultaneously, a wealth of computational methods and tools have emerged for uncovering how protein dynamics affects ligand binding events and allosteric communication at the molecular scale.

In this Research Topic, a total of 20 works has been compiled that will be introduced here based on the diverse computational approaches employed including molecular dynamics (MD) simulations, elastic network models (ENM), hybrid and integrated methods, and protein structure networks (PSN).

MD simulation is the most popular computational method used in complementing experimental techniques as it captures the behavior of proteins in full atomistic detail for understanding binding and allosteric events (Hollingswort and Dror, 2018), as well as molecular aspects of diseases and their treatments. Khan et al., performed an integrated computational study on the estrogen receptor alfa (ERα), which have been observed to be recurrent in metastatic breast cancer patients. The impact of experimentally-reported ERα polymorphisms was studied using techniques such as mCSM stability and binding affinity analysis (Pires et al., 2013) and MD simulations for revealing the dynamical effects on receptor structure. Another cancer type, Glioblastoma (GBM), is the most common and aggressive intracranial malignant brain tumor. Histone deacetylase 1 (HDAC1) is a promising target for therapy of GBM, and macrocyclic peptides have gained great attention due to their remarkable inhibitory selectivity on HDAC1. Zhang et al., employed molecular modeling approaches including molecular docking and MD simulation, along with protein-ligand interaction fingerprints and per-residue binding free energy analysis to explore the binding of a typical macrocyclic peptide FK228 to both HDAC1 and HDAC6. The blockade of programmed death receptor 1 (PD-1) has become a promising therapeutic approach in cancer immunotherapy. In Liu et al.'s work, the binding features of PD-1 with Nivolumab, a humanized IgG4 antibody approved by the US FDA, were investigated using MD simulations. The computational analysis suggested that the N-terminal loop of PD-1 serves as an important gatekeeper for the anti-PD-1 antibody binding, which might be a potential target for anti-PD-1 antibody design.

In the brief research report by Du et al., the structure of Oncostatin M and Receptor (OSM–OSMR) complex was generated as a potential therapeutic target for inflammatory bowel disease. Eight “hot spots” residues and six potential binding sites at the OSM-OSMR interface were predicted using computational alanine scanning and FTMap (Kozakov et al., 2015) analysis, which might be useful to guide further experimental studies and drug design. The article from Shi et al., compared the binding behaviors of four triazole-based inhibitors to sterol 14α demethylase enzyme (CYP51), and identified potential key binding sites. Besides, some possible tunnel pathways of the inhibitors in these CYP51-inhibitor complexes were proposed. Using MD simulations, Du et al. gave a detailed analysis of thermostability factors of barley limit dextrinase, including eight salt-bridges.

Dudas et al. used the hybrid MDeNM (Costa et al., 2015) method that combines MD simulations with classical normal mode analysis for efficient exploration of the conformational space of full-length K-Ras4B. Unbiased conformational sampling was carried out for the GDP- and GTP-bound states to determine the interaction details between a flexible tail-like hypervariable region (HVR) and the catalytic domain of K-Ras4B. The results elucidate the molecular details of a population shift mechanism between the autoinhibited state of the catalytic domain (GDP-bound) and its active state (GTP-bound). Salawu introduced DESP method by combining MD simulations and deep neural networks for enhanced sampling of conformation spaces. Guzel et al. studied allosteric signaling and communication pathways in the exit tunnel of the bacterial ribosome. Due to the huge size of this supramolecular machine, they used coarse-grained MD simulations (RedMD) (Górecki et al., 2009) and an ENM-based hybrid method (ClustENM) (Kurkcuoglu et al., 2016) for conformer generation. Suboptimal pathways based on the contact topology of conformers elucidated the allosteric signaling in the ribosomal tunnel.

Normal mode analysis based on ENM facilitates the study of protein dynamics and allosteric effects in a high-throughput manner (Krieger et al., 2020). Ayyildiz et al., integrated ENM-based methods with computational solvent mapping and sequence/structural alignments for identifying potential allosteric sites. By applying the method to three glycolytic enzymes, the specific interface regions connecting the subunits were predicted as promising target sites for allosteric regulation and species-specific drug design. Two widely used ENMs- Gaussian (GNM) (Bahar et al., 1997) and anisotropic network model (ANM) (Atilgan et al., 2001) - were used by Khairallah et al., to investigate the collective motions of the 6-pyruvol tetrahydropterin synthase (PTPS) enzyme and its allosteric properties. In particular, large fluctuations of the N-terminal domain and its allosteric role were discussed in the context of infectious disease treatment.

Abdizadeh et al. used an ENM-based perturbation response scanning (PRS) (Atilgan and Atilgan, 2009) to identify the residues that trigger dissociation between interacting protein pairs. Based on a set of 25 protein complexes, such residues were identified to be located either at regions with large conformational changes or at parts of the protein that are structurally unaffected. Interestingly, the interfacial residues were responsible for the dissociation in only a few of the complexes. Furthermore, they categorized four modes of dissociation based on PRS and electrostatic effects. Jernigan et al. proposed two ENM-based approaches for designing peptide-based inhibitors using the influenza protein hemagglutinin (HA) as the case study. Based on available experimental structures conformationally most variable region of HA was identified as a potential target for diverse ligands. Furthermore, the empirical contact potentials including an ENM-based entropy term were found successful in ranking the free energies of peptide/proteins designed against HA.

Topological description of protein structures has become a popular tool to quantify protein structures and dynamics (Liang et al., 2020). By means of joint dynamical/topological description of SARS-COV2 spike protein, Halder et al., gave a thorough presentation on the network-dynamical systemic approach to protein function. In addition, their work highlighted the advantages of the side-chain network analysis in studying subtle conformational changes with an emphasis related to allostery. Gosu et al., used PSN to study the MD ensemble of Myeloid differentiating factor 88 (Myd88) and AlloSigMA (Guarnera et al., 2017) to describe allosteric effects of S34Y and R98C variants. These two mutations were shown not only to induce a large conformational change of Myd88, but also influence the interaction with other death domains in TLR downstream signaling. Wang et al., combined PSN with some structural dynamics approaches such as molecular docking, MD simulations, and free energy calculation to study specific interactions between crocin and tubulin. Their results pointed to a common residue motif (val175-Xxx176-Asp177) that could serve as a potential binding site. Yan et al. designed the ANCA webserver for constructing and analyzing PSN for interpretation of functional residues and allosteric regulation.

Three review papers complete our Research Topic in experimental and computational prospects. Isothermal titration calorimetry (ITC) is a technique that measures the thermodynamics of binding reactions and reaction kinetics. The review by Wang et al., give a broad overview of the use of ITC to measure the strength, mode, and association/dissociation kinetics of enzyme inhibitors, as well as its potential applications on allostery and drug design. Jaffe describes the morpheeins model for allosteric regulation by focusing on porphobilinogen synthase. This model characterizes the dynamic nature of quaternary structures/assemblies formed by homo-multimeric proteins with its implications/applications for drug discovery. Lastly, Verkhivker et al., provide a comprehensive overview on the state-of-the-art approaches that advance quantitative characterization of allosteric mechanisms in proteins, including experiment-guided Markovian models, simulation-based multiscale approaches and machine learning. These frontier technologies not only provide tools for the studying of molecular basis of allosteric mechanisms, but also help the discovery of allosteric modulators for therapeutically important protein targets.

## Author Contributions

All authors listed have made a substantial, direct and intellectual contribution to the work, and approved it for publication.

## Conflict of Interest

The authors declare that the research was conducted in the absence of any commercial or financial relationships that could be construed as a potential conflict of interest.

## References

[B1] Atilgan A. R. Durell S. R. Jernigan R. L. Demirel M. C. Keskin O. Bahar I. (2001). Anisotropy of fluctuation dynamics of proteins with an elastic network model. Biophys. J. 80, 505–515. 10.1016/S0006-3495(01)76033-X 11159421 PMC1301252

[B2] Atilgan C. Atilgan A. R. (2009). Perturbation-response scanning reveals ligand entry-exit mechanisms of ferric binding protein. PLoS Comput. Biol. 5:e000544. 10.1371/journal.pcbi.1000544 19851447 PMC2758672

[B3] Bahar I. Atilgan A. R. Erman B. (1997). Direct evaluation of thermal fluctuations in proteins using a single-parameter harmonic potential. Fold Des. 2, 173–181. 10.1016/S1359-0278(97)00024-2 9218955

[B4] Costa M. G. S. Batista P. R. Bisch P. M. Perahia D. (2015). Exploring free energy landscapes of large conformational changes: molecular dynamics with excited normal modes. J. Chem. Theory Comput. 11, 2755–2767. 10.1021/acs.jctc.5b00003 26575568

[B5] Górecki A. Szypowski M. Długosz M. Trylska J. (2009). RedMD-redcuded molecular dynamics. J. Comput. Chem. 30, 2364–2374. 10.1002/jcc.21223 19247989

[B6] Guarnera E. Tan Z. W. Zheng Z. Berezovsky I. N. (2017). AlloSigMA: allosteric signaling and mutation analysis server. Bioinformatics 33, 3996–3998. 10.1093/bioinformatics/btx430 29106449

[B7] Gunesakaran K. Ma B. Nussinov R. (2004). Is allostery an intrinsic property of all dynamic proteins? Proteins Struct. Funct. Bioinform. 57, 433–443. 10.1002/prot.20232 15382234

[B8] Hollingswort S. A. Dror R. O. (2018). Molecular dynamics simulation for all. Neuron 99, 1129–1143. 10.1016/j.neuron.2018.08.011 30236283 PMC6209097

[B9] Kozakov D. Grove L. E. Hall D. R. Bohnuud T. Mottarella S. E. Luo L.. (2015). The FTMap family of web servers for determining and characterizing ligand-binding hot spots of proteins. Nat. Protoc. 10, 733–755. 10.1038/nprot.2015.043 25855957 PMC4762777

[B10] Krieger J. M. Doruker P. Scott A. L. Perahia D. Bahar I. (2020). Towards gaining sight of multiscale events: utilizing network models and normal modes in hybrid methods. Curr. Opin. Struct. Biol. 64, 34–41. 10.1016/j.sbi.2020.05.013 32622329 PMC7666066

[B11] Kurkcuoglu Z. Bahar I. Doruker P. (2016). ClustENM: ENM-based sampling of essential conformational space at full atomic resolution. J. Chem. Theory Comput. 12, 4549–4562. 10.1021/acs.jctc.6b00319 27494296 PMC5088496

[B12] Liang Z. Verkhivker G. M. Hu G. (2020). Integration of network models and evolutionary analysis into high-throughput modeling of protein dynamics and allosteric regulation: theory, tools and applications. Brief Bioinformat. 21 815–835. 10.1093/bib/bbz029 30911759

[B13] Pires D. E. Ascher D. B. Blundell T. L. (2013). mCSM: predicting the effects of mutations in proteins using graph-based signatures. Bioinformatics 30, 335–342. 10.1093/bioinformatics/btt691 24281696 PMC3904523

[B14] Zhang Y. Doruker P. Kaynak B. Zhang S. Krieger J. Li H.. (2020). Intrinsic dynamics is evolutionarily optimized to enable allosteric behavior. Curr. Opin. Struct. Biol. 62, 14–21. 10.1016/j.sbi.2019.11.002 31785465 PMC7250723

